# Antibacterial Textile Coating Armoured with Aggregation-Induced Emission Photosensitisers to Prevent Healthcare-Associated Infections

**DOI:** 10.3390/molecules29061209

**Published:** 2024-03-08

**Authors:** Resmarani Sahu, Neethu Ninan, Ngoc Huu Nguyen, Jianzhong Wang, Krasimir Vasilev, Vi Khanh Truong, Youhong Tang

**Affiliations:** 1 College of Medicine and Public Health, Flinders University, Bedford Park, Adelaide, SA 5042, Australia; sahu0011@flinders.edu.au (R.S.); neethu.ninan@flinders.edu.au (N.N.); ngochuu.nguyen@flinders.edu.au (N.H.N.); krasimir.vasilev@flinders.edu.au (K.V.); 2 Institute for NanoScale Science and Technology, Medical Device Research Institute, College of Science and Engineering, Flinders University, Bedford Park, Adelaide, SA 5042, Australia; wjzd2005@163.com; 3 College of Veterinary Medicine, Jilin Agricultural University, Changchun 130118, China

**Keywords:** aggregation-induced emission photosensitisers, healthcare-associated infections, plasma coating, antibacterial, textile

## Abstract

In the quest to curtail the spread of healthcare-associated infections, this work showcases the fabrication of a cutting-edge antibacterial textile coating armoured with aggregation-induced emission photosensitisers (AIE PS) to prevent bacterial colonisation on textiles. The adopted methodology includes a multi-step process using plasma polymerisation and subsequent integration of AIE PS on their surface. The antibacterial effectiveness of the coating was tested against *Pseudomonas aeruginosa* and *Staphylococcus aureus* after light irradiation for 1 h. Furthermore, antibacterial mechanistic studies revealed their ability to generate reactive oxygen species that can damage bacterial cell membrane integrity. The results of this investigation can be used to develop ground-breaking explanations for infection deterrence, principally in situations where hospital fabrics play a critical part in the transmission of diseases. The antibacterial coating for textiles developed in this study holds great promise as an efficient strategy to promote public health and reduce the danger of bacterial diseases through regular contact with fabrics.

## 1. Introduction

Over the previous years, there have been recurrent occurrences of epidemics, including tuberculosis, influenza, pneumonia, gastroenteritis, and COVID-19, that have resulted in significant mortality globally [1,2]. These outbreaks have heightened the scope of the spread of infections from healthcare settings, affecting patients, healthcare workers, etc. [3]. Healthcare-associated infections (HAIs) are thus global health concerns impacting millions of people [4]. According to the World Health Organization (WHO), among every 100 patients admitted to hospitals, 15 patients in middle- and low-income countries and 7 patients in high-income countries contract HAIs. An average of one out of ten patients dies after acquiring HAI. Contaminated hospital fabrics (bedding, carpets, curtains, towels, etc.) are the principal sources of the spread of diseases [5]. Even after periodic cleaning, the clothing of healthcare workers has been found to have a substantial microbial load, and 92% of curtains used in healthcare settings are contaminated within a week after thorough cleaning [6]. In this context, the synthesis of advanced textile coating is of the utmost necessity to prevent the spread of infections. Researchers are investigating various approaches to load antimicrobial agents within fabrics to control the spread of HAIs [7,8,9]. The recent mainstream stratagems include the physical loading of antimicrobial agents such as polypyrrole, quarternary ammonium compounds, chitosan, polyhexamethylene biguanide, natural dyes, N-halamide, copper, silver, etc., into textiles [7,10,11,12]. However, the main drawback of physical adsorption is the likelihood of detachment from the surface [13]. Covalent immobilization overwhelms the instability issues and the leaching of antimicrobial agents attached to the surface [14]. Nevertheless, the chemistry behind this method is multifaceted, substrate-dependent, and requires a significant amount of solvents. Versatile and precise surface engineering is possible using plasma polymerisation, a substrate-independent technique that can deposit a thin film of plasma polymer on virtually any substance [13,15]. This technique is flexible over varied substrates, unlike other methods such as self-assembled monolayers and layer-by-layer techniques. Plasma polymerisation can generate a range of reactive functional groups at the interface of a material that can effectively bind nanoparticles and other molecules, which showcases the adaptability of this technique. The absence of solvents, minimal input of precursor, lack of organic wastes, and environmentally friendly nature are attractive features of this technique [13,15]. Polyoxazoline (OX)-based coatings were chosen for this study due to their low fouling characteristics, biocompatibility, stability, and capacity to bind nanomaterials and bioactive molecules [13,15].

Photodynamic therapy is an important technique for the treatment of many diseases. It contains three key elements: oxygen, light, and photosensitizers. The photosensitizer is activated in the presence of light and converts tissue oxygen to reactive oxygen species (ROS) such as singlet oxygen [^1^O_2_], peroxyl radical [H_2_O_2_], superoxide anion radical [O^2−^], and hydroxyl radical [HO•], which can damage the bacterial cell membrane [16]. There are several photosensitizers currently emerging in the biomedical research sector for various applications, such as 5-aminolevulinic acid (ALA), hypericin, hematoporphyrin derivatives (HpD), foscan, aluminium (III) phthalocyanine tetrasulfonate chloride (AlPcS4), photofrin, fospeg, etc. These photosensitizers have great absorption spectra along with excellent tissue selectivity [9]. However, they have some limitations, such as having very limited tissue targeting capabilities, reduced ROS generation because of the aggregation quenching effect, and decreased stability. Furthermore, some of these photosensitizers express high cytotoxicity, less biodegradability, complicated pharmacokinetics, and poor repeatability.

Aggregation-induced emission photosensitizers (AIE PS) are mesmerising groups of materials in the nano-range that exhibit higher luminescence in their aggregated state, and not in their molecular state [17,18,19,20,21]. AIE PS provide excellent stability, along with therapeutic and fluorescence-imaging contrast agents, for many diagnoses and therapies [16]. These AIE probes were created for biological and biomedical uses, including the detection of bacteria, tracking of cells, imaging of organelles, biomolecule labelling, tumour imaging and diagnostics, etc. [16]. The main drawback of traditional fluorophores is fluorescence quenching in aggregated states, which AIE PS overcome [22]. The high emission efficiency, target specificity and biocompatibility of AIE PS can be harnessed for antibacterial applications [23]. TPAQ-PF6 (Triphenylamine quinolinium hexafluorophosphate) is the AIE PS used in this study [24]. This cationic moiety was able to generate reactive oxygen species (ROS) to disrupt bacterial cell membranes. TPAQ-PF6 is composed of an electron donor group (triphenylamine) and an electron-withdrawing group (quinolinium hexafluorophosphate), forming a donor–acceptor (D-A) configuration [24]. They can generate ROS, which disrupt bacterial cellular organelles. Unlike antibiotics, which target specific metabolic pathways, these ROS-generating materials can overcome multi-drug-resistant bacteria that cause HAIs [25]. Integration of these molecules on plasma coatings will improve their antibacterial activities.

In this study, we prepared OX-based plasma coatings onto which rationally designed cationic derivatives of AIE PS were immobilised. Their detailed physiochemical characterisation was conducted. Their antibacterial activities were explored on medically relevant pathogens of Gram-positive *Staphylococcus aureus* and Gram-negative *Pseudomonas aeruginosa*. Their antibacterial mechanisms were studied in depth using ROS generation assays.

## 2. Results and Discussion

**Scheme 1:**
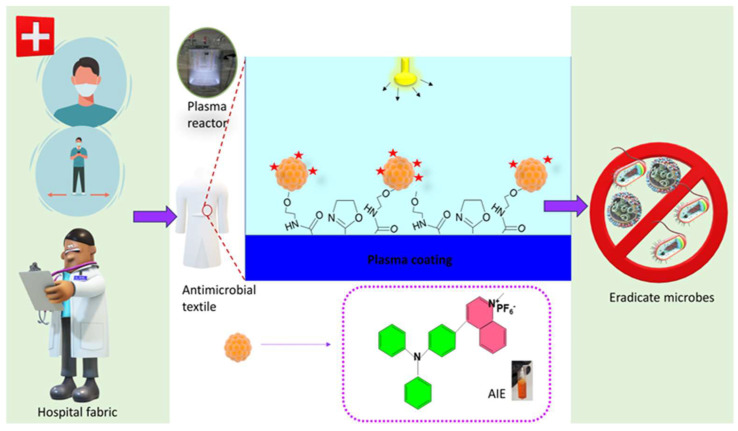
Schematic representation of design and development of polymeric fabric coating using plasma polymerisation and AIE PS.

**Figure 1:**
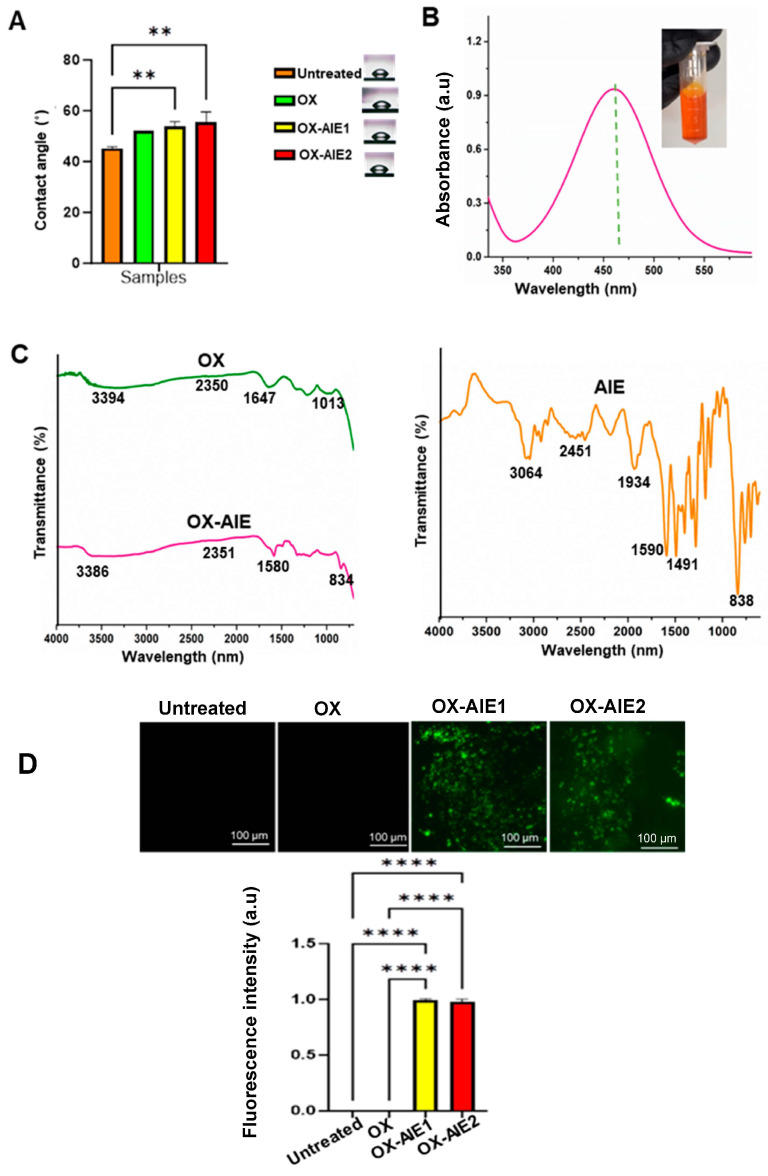
(**A**) Water contact angle and drop images of untreated sample, OX, OX-AIE1, and OX-AIE2. (**B**) UV absorption spectrum of AIE solution (TPAQ-PF_6_). (**C**) FTIR spectra of OX, OX-AIE, and AIE (TPAQ-PF_6_). (**D**) Binding studies from fluorescence images and fluorescence intensity profiles of untreated sample, OX, OX-AIE1, and OX-AIE2. The * shows a significant difference compared to the OX according to 1-way ANOVA, with mean ± SD, *n* = 3, ** *p* < 0.01, **** *p* < 0.0001.

The experimental strategy to fabricate antibacterial coating for textiles using plasma polymerisation and AIE PS is shown in Scheme 1. First, an OX-based plasma polymer was deposited on the surface of substrates. The intact oxazoline rings of OX are known to interact with nanoparticles, antibodies, proteins, and other biomolecules. The thickness of the OX coating was around 24.3 ± 2.0 nm, as assessed using ellipsometry. Meanwhile, AIE PS was dissolved in an ethanol–water mixture (0.46 and 0.33 mg/mL) and exhibited a unique UV absorption peak around 460 nm (Figure 1B). The OX-coated samples were incubated in two different concentrations (0.46 and 0.33 mg/mL) of AIE PS for 24 h, thoroughly washed with water, and dried with nitrogen gas. The samples attained an orangish-yellow colour and were named OX-AIE1 and OX-AIE2. The binding of AIE on OX-coated surfaces was verified using fluorescence microscopy. There was hardly any fluorescence on uncoated and POX-coated surfaces. However, OX-AIE1 and OX-AIE2 exhibited fluorescence at the emission wavelength of AIE PS at 595 nm. However, no significant differences were noticed in their fluorescence intensities (Figure 1D).

Wettability has a pivotal role in dictating the adhesion of bacteria on fabric. It is governed by various factors, including topography and surface chemistry. OX has a static water contact angle of around 52 ± 2.3° (Figure 1A). After the immobilisation of AIE PS, the water contact angle of OX-AIE1 and OX-AIE2 increased. This was attributed to the hydrophobic groups in AIE PS. Hydrophobic textiles have attained momentous attention because they avoid dirt accumulation, increase the lifespan of fabric by preventing degradation in water, and avert bacterial accumulation.

Next, the presence of AIE PS on plasma-coated surfaces was verified from FTIR spectra. The chemical functionalities presented in the FTIR spectrum of OX included the C^_^O group at 1013 cm^−1^ and the C=N group at 1647 cm^−1^ due to oxazoline rings in OX (Figure 1C). The peak at 2350 cm^−1^ was attributed to isocyanate or nitrile groups in OX. AIE PS contain several characteristic peaks, including aromatic rings at 1491 cm^−1^, nitrile groups at 2451 cm^−1^, N-H bending at 1590 cm^−1^, and a unique peak in the fingerprint region at 838 cm^−1^ from PF_6_ groups. Interestingly, all of these typical peaks of AIE PS were found in OX-AIE, which confirmed their successful immobilisation. Additionally, the shift of the peaks from 1647 cm^−1^ to 1580 cm^−1^ in the case of OX-AIE may have been due to the interaction of oxazoline groups of OX with the nucleophilic sites in AIE PS. 

**Figure 2:**
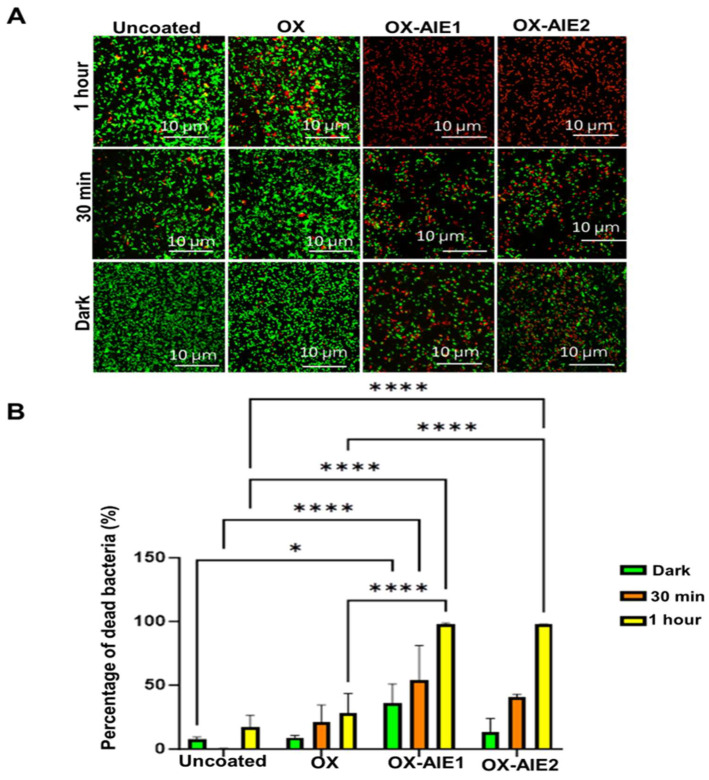
(**A**) Confocal live/dead stained images and (**B**) viability of *P. aeruginosa* of untreated sample, OX, OX-AIE1, and POX-AIE2 under dark conditions and after light exposure for 1 h and 30 min with mean ± SD, *n* = 3. * *p* < 0.05, **** *p* < 0.0001. The * shows a significant difference compared to the OX according to 1-way ANOVA.

**Figure 3:**
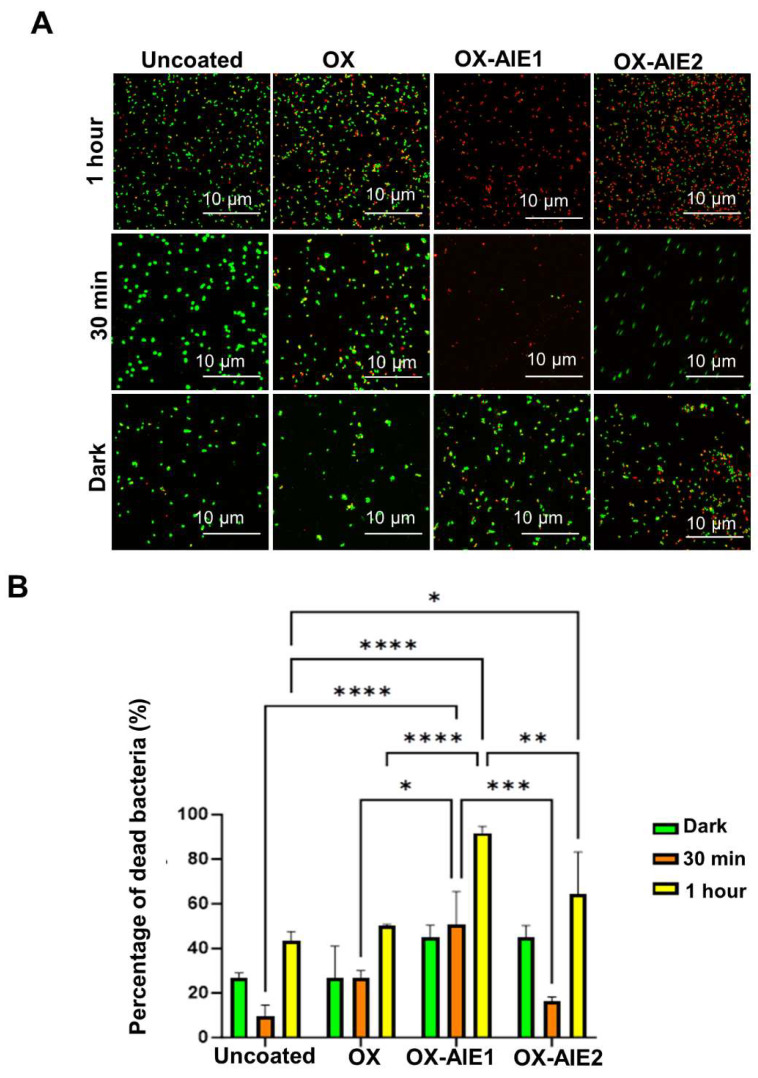
(**A**) Confocal live/dead stained images and (**B**) viability of *S. aureus* of untreated sample, OX, OX-AIE1, and POX-AIE2 under dark conditions and after light exposure for 1 h and 30 min with mean ± SD, *n* = 3. * *p* < 0.05, ** *p* < 0.01, *** *p* < 0.001, **** *p* < 0.0001. The * shows a significant difference compared to the OX according to 1-way ANOVA.

The bactericidal activity of the plasma-modified surfaces was evaluated using a live dead assay under dark conditions and after light exposure for 30 min and 1 h. *S. aureus* and *P. aeruginosa* were the typical Gram-positive and Gram-negative bacteria chosen for the study, as they are mostly found in contaminated hospital fabrics and are involved in the transmission of HAIs [26]. Figure 2 and Figure 3 demonstrate that the viability of both *S. aureus* and *P. aeruginosa* was least affected on uncoated and OX-coated surfaces. The bactericidal effect after light irradiation was significantly higher than in dark conditions, as demonstrated by the presence of more dead cells (stained red). The killing percentages of *S. aureus* on OX-AIE1 and OX-AIE2 were 91.0 ± 3.0% and 64.0 ± 18.0%, respectively. The percentages of dead *P. aeruginosa* on OX-AIE1 and OX-AIE2 were 97.0 ± 1.2% and 97.0 ± 0.1%. *P. aeruginosa* were more susceptible to the antibacterial effects than *S. aureus,* as the cationic AIE PS employed in this study (TPAQ-PF_6_) had good affinity to Gram-negative bacteria, whose peptidoglycan layers are thinner when compared to Gram-positive bacteria. OX-AIE1 had the highest bactericidal activity when compared to all others. The results of the colony-forming unit assay further verified that OX-AIE1 exhibited the highest antibacterial ability among all others, and it will be used for further studies (Figure S1).

**Figure 4:**
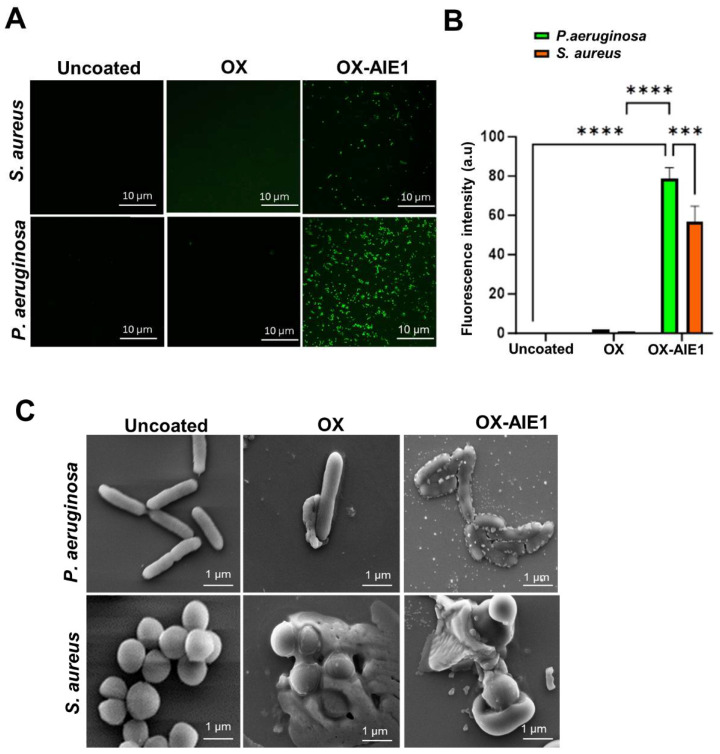
(**A**) Intracellular ROS generation; (**B**) fluorescence intensity profile; and (**C**) SEM images of *S. aureus* and *P. aeroginosa* exposed to untreated sample, OX, and OX-AIE1. (*n* = 3, all data represented as mean ± SD, *** *p* < 0.001, **** *p* < 0.0001). The * shows a significant difference compared to the OX according to 1-way ANOVA.

The possible mechanisms of antibacterial action were explored using ROS assays, membrane potential, and illustrative SEM images. ROS are highly reactive molecules that play innumerable roles in living cells [27]. They are generated as a result of the response of bacteria to oxidative stress. ROS generation in bacteria is identified using a green fluorescent dye, dichlorofluorescein (DCF). A higher amount of intracellular ROS generation is demonstrated by an increase in the intensity of green fluorescence. In this study, bacterial strains (*P. aeruginosa* and *S. aureus*) exposed to OX-AIE1 presented increased levels of ROS compared to OX (Figure 4A). From the fluorescent intensity data, it is evident that more ROS were generated in the OX-AIE1 samples exposed to *P. aeruginosa* compared to *S. aureus* (Figure 4B). This was in agreement with the live/dead assay results discussed previously.

The changes in the structure of bacterial cells (*S. aureus* and *P. aeruginosa)* when untreated and when treated with OX and OX-AIE1 were visualised using SEM images (Figure 4C). Normal *P. aeruginosa* and *S. aureus* adopted rod and spherical shapes, respectively. The bacterial cells were robust and healthy for the untreated samples. Wrinkled and distorted morphology was observed in the case of bacteria treated with OX-AIE1. The cell integrity was affected, causing cell membrane damage. OX-AIE1 showed strong bactericidal action against both *P. aeruginosa* and *S. aureus.* The cell permeability experiment confirmed that OX-AIE1 resulted in damage to the cellular architecture and swelling of all intracellular components compared to OX (Figure S2).

**Figure 5:**
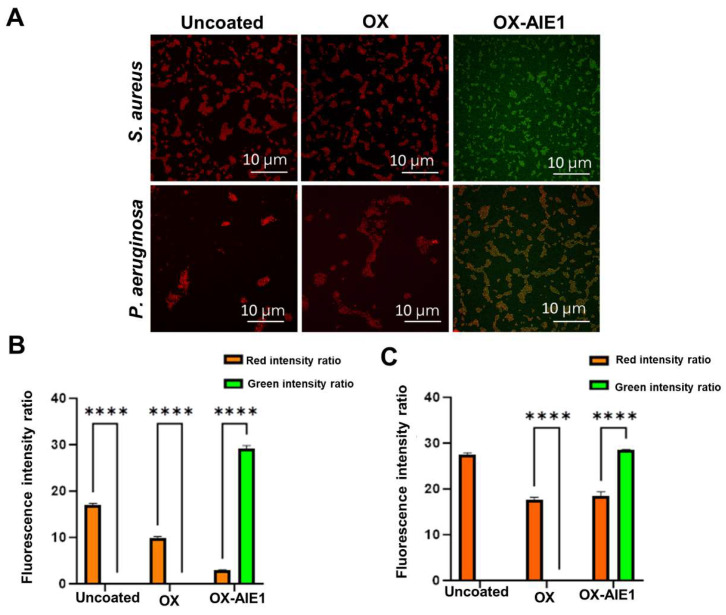
(**A**) Analysis of membrane potential from confocal images and (**B**,**C**) fluorescence intensity ratio profile of uncoated sample, OX, and OX-AIE1 treated with (**B**) *S. aureus* and (**C**) *P. aeroginosa.* (*n* = 3, all data represented as mean ± SD, **** *p* < 0.0001). The * shows a significant difference compared to the OX according to 1-way ANOVA.

Additionally, after exposing bacteria to AIE PS immobilized materials, we observed changes in their membrane potential using probe DiO. Cell membrane potential, or the electric potential surrounding the cell membrane, is what gives the cell its free energy to perform all chemical and mechanical functions [28]. It influences the ability of bacteria to divide, as well as their metabolism, intake of nutrients, and survival [29]. Therefore, by using antibiotics and other antibacterial treatments to target this location, the bacterial cells can be destroyed. Figure 5 reveals that OX-AIE1 had a significant impact on the membrane potential of *S. aureus* and *P. aeruginosa* bacterial cells at a lower concentration (10 µg/mL). Moreover, *S. aureus* showed a greater degree of membrane alteration following exposure to OX-AIE1.

## 3. Materials and Methods

### 3.1. Materials

The Aggregation-Induced Emission Institute from China provided the AIE PS required for this study. Ultrapure water was attained from a Milli Q system (Millipore Milli-Q Academic, McKees Rocks, PA, USA). Dulbecco’s Modified Eagle’s Medium (DMEM), 2-methyl-2-oxazoline, and phosphate-buffered saline (PBS) were purchased from Sigma-Aldrich, Bayswater, Australia. Ethanol and acetone were purchased from ChemSupply, Gillman, Australia. Streptomycin and penicillin were bought from Life Technologies. Tryptone soy broth (TSB) and foetal bovine serum (FBS) were obtained from Oxoid and Thermo Scientific, Scoresby, Australia. 2′,7′-dichlorodihydrofluorescein diacetate (DCF) and a live/dead viability kit (BacLight^TM^) were bought from Thermo Fischer Scientific, Scoresby, Australia. Silicon wafers and glass coverslips (G400-13) were purchased from ProSciTech, Kirwan, Australia. For the anti-bacterial experiments, *Pseudomonas aeruginosa* (*P. aeruginosa*, ATCC 15692) and *Staphylococcus aureus* (*S. aureus*, ATCC 25923) were cultured.

### 3.2. Methods

#### 3.2.1. Preparation of Plasma-Functionalised Surfaces

A specially designed bell chamber plasma reactor was used to coat the substrates (1.0 cm^2^ silicon wafers and 1.0 cm diameter glass coverslips) with a thin coating of plasma polymer after they had been properly cleaned with acetone and ethanol and dried using nitrogen flow. For five minutes, the substrates were exposed to air plasma at a power of 50 W and a pressure of 1 × 10^−1^ mbar. These substrates were covered with thin coatings of OX for two minutes at a pressure of 1.3 × 10^−1^ mbar and 50 W. Vacuum-sealed containers were used to store the coated substrates. 

#### 3.2.2. Surface Immobilisation Using AIE PS

A combination of ethanol and water was used to generate two distinct concentrations of AIE PS (TPAQ-PF6): AIE1 was 0.46 mg/mL and AIE2 was 0.33 mg/mL. After being submerged in the solution, the POX-coated substrates were incubated for 24 h. Following the immobilisation time, the samples underwent three MilliQ water washes and nitrogen gas drying before being given the respective names OX-AIE1 and OX-AIE2.

#### 3.2.3. Ellipsometry

Silicon wafers covered with OX were measured for thickness using a photographic ellipsometer from SENresearch, SENTECH, Berlin, Germany. During the analysis, the 2 nm SiO_2_ layer on the silicon wafer substrate was removed. The working parameters of the ellipsometer were established, and the substrates were placed on the platform. The layer’s thickness was assessed using SpectraRay/4 software (https://www.sentech.com/e n/SENresearch__219/, accessed on 29 January 2024). 

#### 3.2.4. Water Contact Angle

This study determined the static water contact angle of the uncoated sample, OX, OX-AIE1, and OX-AIE2. The RD-SDMO2 goniometer’s syringe was used to place a 2.0 µL water droplet on top of the substrate, and pictures were taken. Furthermore, by measuring the contact angle with the ImageJ software’s drop snake analysis plugin, the types (hydrophobic/hydrophilic) of the functionalized surfaces were ascertained.

#### 3.2.5. UV Visible Spectroscopy

Using a Perkin Lambda 350 spectrophotometer, the UV-visible spectra of the TPAQ-PF6 (AIE PS) solution was acquired across the wavelength range of 200–900 nm.

#### 3.2.6. Fourier Transform Infrared Microscopy (FTIR)

Using Perkin Elmer FTIR, potential interactions between OX and AIE PS were examined throughout a wavenumber range of 400 to 4000 cm^−1^.

#### 3.2.7. Fluorescent Microscope

Olympus IX83 Inverted Fluorescence was used to capture the pictures (excitation wavelengths of 475–490 nm and emission wavelength of 595 nm) and determine the fluorescence intensity of the untreated sample, OX, OX-AIE1, and OX-AIE2. Data analysis was performed using Zeiss Zen 3.8 software.

#### 3.2.8. Scanning Electron Microscopy (SEM)

Using an FEI Inspect F50 Field Emission SEM, the samples’ morphology was examined. Using 4% glutaraldehyde for 45 min, the biological samples were first fixed. The samples underwent a 10-min dehydration period using a graded ethanol series of 30, 50, 70, 80, 90, and 100%. After the samples were dehydrated, they were dried with nitrogen gas and coated with platinum using an Ion Sputter Coater (TB-SPUTTER) obtained from Quorum Technologies in the Lewes, UK.

#### 3.2.9. Live/Dead Antibacterial Assay

*S. aureus* and *P. aeruginosa* were inoculated in TSB and allowed to incubate for the entire night at 37 °C. Following the mid-log phase, 1 × 10^6^ colony-forming units (CFU/mL) of the bacterial culture were diluted. The diluted bacterial solution were incubated separately with silicon wafers functionalized with the untreated sample, OX, OX-AIE1, and OX-AIE2. After being incubated for 1 h, they were exposed to light at 40 mW/cm^2^. Using STYO9 and propidium iodide (PI) from the LIVE/DEAD BacLightTM Viability Kit (Molecular Probes, Invitrogen, Waltham, MA, USA), the silicon wafers were stained for 10 min in the dark while being rinsed with PBS. A Zeiss LSM 880 confocal microscope (Zeiss, Oberkochen, Germany) was then used to visualise them.

#### 3.2.10. Colony Enumeration

A bacterial suspension of *S. aureus* and *P. aeruginosa* (1 × 10^8^ CFU/ mL, 10 µL) was cultured for 1 h at 37 °C on TPAQ-PF6 immobilised plasma-coated samples. The samples were thereafter subjected to either 1 h of exposure to 40 mW/cm^2^ of white light or to complete darkness. Next, the suspension underwent a 5-min shakedown and cleaning cycle. A nutrient broth agar plate was then used to plate the suspension after it had been serially diluted using the proper folds. After 20 h at 37 °C, they were incubated. The next day, the colonies were tallied and counted again.

#### 3.2.11. Reactive Oxygen Species (ROS) Assay

After 1 h of light exposure, the silicon wafers that had been modified by plasma (untreated sample, OX, and OX-AIE1) were incubated independently with 10^8^ CFU/mL of *S. aureus* and *P. aeruginosa*. After applying 10 μL of 2′,7′-Dichlorofluorescein dye (in PBS), the wafers were rinsed with PBS. Under dark circumstances, they were incubated for 45 min at 37 °C. A Zeiss LSM 880 confocal microscope from Germany was used to visualise them.

#### 3.2.12. Membrane Potential

The AIE PS immobilised OX-polymerised substrate was treated with *S. aureus* and *P. aeruginosa* under 1 h of light irradiation. The uncoated and OX-coated samples were considered as positive and negative controls. Following a PBS wash, the samples were stained for 10 min using the DiO/DPA membrane potential detection kit (Invitrogen, ThermoFisher, Waltham, MA, USA). Green and red fluorescence were measured using confocal laser microscopy (Zeiss LSM 880, Oberkochen, Germany) at excitation/emission wavelengths of λ_abs_ = 484 nm for DiO and λ_abs_ = 406 nm for DPA. Using Zen (Black Edition) and ImageJ v1.53a (NIH, Bethesda, MD, USA) for image processing, the intensity of the red and green fluorescence was measured, and their ratio was calculated.

#### 3.2.13. Membrane Integrity

With 1 h of light irradiation, the AIE PS immobilised plasma-polymerised substrate was immersed in the bacterial solutions of *S. aureus* and *P. aeruginosa*. It was then stained with CellBrite^®^ Fix 640 (Biotium, Fremont, CA, USA). Briefly, the bacterial cultures were incubated with CellBrite^®^ Fix 640 for 2 h. Following staining, the bacterial cultures were imaged using a confocal microscope. Images were acquired using an excitation wavelength of 638 nm and an emission wavelength of 667 nm, which are optimal for CellBrite^®^ Fix 640. The acquired images were analysed using standard image processing Zen software (https://www.zeiss.com/microscopy/en/products/software/zeiss-zen-lite.html, accessed on 29 January 2024). The integrity of the bacterial membranes was assessed based on the uniformity and intensity of the CellBrite^®^ Fix 640 staining.

## 4. Conclusions

Recently, the demand for hospital fabrics with active antibacterial ingredients based on nanotechnology has significantly increased. In this study, we explored the use of plasma coating to develop textiles with surface-anchored AIE PS for the photodynamic killing of bacteria and, thereby, to control the transmission of diseases. Primarily, thin films of plasma coatings in the range of nanometres were deposited on suitable substrates using a 2-methyl 2-oxazoline precursor. TPAQ-PF6 (or AIE PS) was bound onto these functionalised surfaces at two different concentrations. We anticipated that the nucleophilic sites in AIE PS might interact with oxazoline moieties in OX. From fluorescence microscopic evaluations, we confirmed the presence of AIE PS successfully bound onto OX-coated surfaces. Bactericidal effects were observed on *P. aeruginosa* and *S. aureus* after irradiation by light. The mechanistic studies revealed their ability to generate intracellular ROS in bacteria, which led to cell death. These discoveries unravel novel prospects for the reproducible and sustainable manufacturing of antimicrobial textiles in the health sector.

## Data Availability

Dataset available on request from the authors.

## Supplementary Materials

The following supporting information can be downloaded at: https://www.mdpi.com/article/10.3390/molecules29061209/s1, Figure S1: Count of colony-forming units of *S. aureus* and *P. aeruginosa* treated with AIE PS solution, OX-AIE1, and OX-AIE2; Figure S2: Confocal images representing cell permeability experiments of *S. aureus* and *P. aeruginosa* treated with OX and OX-AIE1.
