# Supplementary material for: Antibacterial Textile Coating Armoured with Aggregation-Induced Emission Photosensitisers to Prevent Healthcare-Associated Infections
Source: Molecules. 2024 Mar 8;29(6):1209. doi: 10.3390/molecules29061209 (PMC10975846; doi:10.3390/molecules29061209)
Supplement: Supplementary file 1 [file molecules-29-01209-s001.zip › molecules-2872738-supplementary.pdf]

Antibacterial Textile Coating Armoured with Aggregation-Induced Emission Photosensitisers to Prevent Healthcare-Associated Infections

Resmarani Sahu [1,2,†], Neethu Ninan [1,†], Ngoc Huu Nguyen [1], Jianzhong Wang [2,3], Krasimir Vasilev [1], Vi Khanh Truong [1,*] and Youhong Tang [2,*]

[1] College of Medicine and Public Health, Flinders University, Bedford Park, Adelaide, SA 5042, Australia; sahu0011@flinders.edu.au (R.S.); neethu.ninan@flinders.edu.au (N.N.); ngochuu.nguyen@flinders.edu.au (N.H.N.); krasimir.vasilev@flinders.edu.au (K.V.)

[2] Institute for NanoScale Science and Technology, Medical Device Research Institute, College of Science and Engineering, Flinders University, Bedford Park, Adelaide, SA 5042, Australia; wjzd2005@163.com

[3] College of Veterinary Medicine, Jilin Agricultural University, Changchun 130118, China

* Correspondence: vikhanh.truong@flinders.edu.au (V.K.T.); youhong.tang@flinders.edu.au (Y.T.); Tel.: +61-8-82012138 (Y.T.)

† These authors contributed equally to this work.

Supplementary Figures

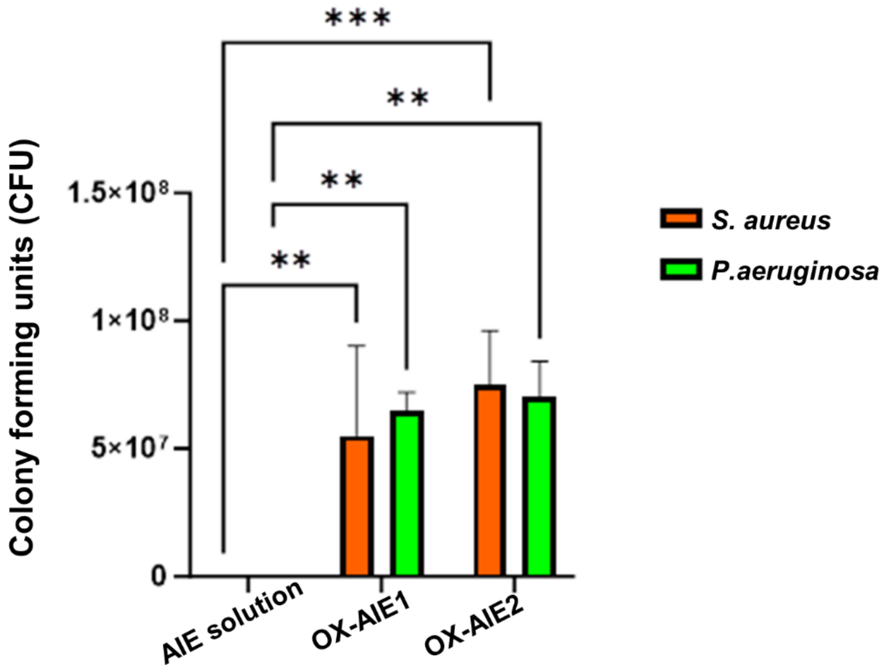

Figure S1. Count of colony-forming units of *S.aureus* and *P.aeruginosa* treated with AIE PS solution, OX-AIE1 and OX-AIE2. Data illustrates mean ± SD, * $p<0.05$, ** $p<0.01$p, *** $p<0.001$,

**** p<0.0001. The * shows a significant difference compared to the AIE solution by 1-way ANOVA.

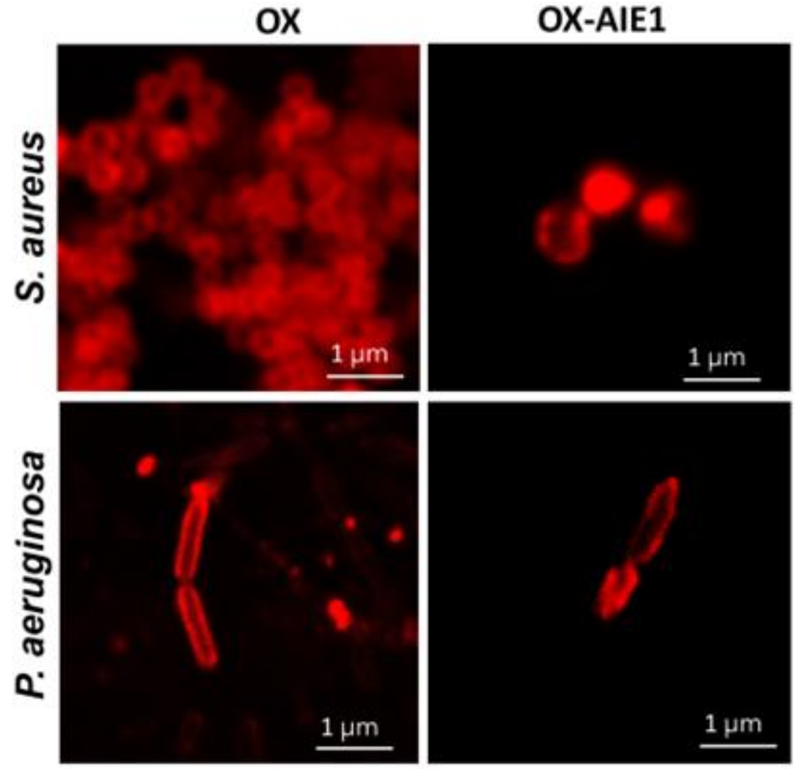

Figure S2. Confocal images representing cell permeability experiments of *S.aureus* and *P.aeruginosa* treated with OX and OX-AIE1.
